# A comparative analysis of cluster based interventions on healthcare-associated infections in a tertiary care hospital in China

**DOI:** 10.3389/fpubh.2025.1599682

**Published:** 2025-06-16

**Authors:** Ya Zou, Chuyu Lao, Ting Fan, Tian Wang, Guanwen Lin, Cuiqiong Fan, Yisui Cen, Yukun Lin, Miao Yang, Congrong Li, Zihuan Li

**Affiliations:** ^1^Department of Infection Prevention and Control, The Affiliated Guangdong Second Provincial General Hospital of Jinan University, Guangzhou, China; ^2^Department of Pharmacy, The First Affiliated Hospital of Sun Yat-sen University, Guangzhou, China; ^3^Department of Prevention and Healthcare, The Affiliated Guangdong Second Provincial General Hospital of Jinan University, Guangzhou, China; ^4^Department of Infection Prevention and Control, Guangdong Second Provincial General Hospital Zengcheng Hospital (Guangdong Hydropower Group Hospital), Guangzhou, China; ^5^Biosafety Laboratory, Zhongshan School of Medicine, Sun Yat-sen University, Guangzhou, China

**Keywords:** cluster-based interventions, healthcare-associated infections, infection control observers team, costs of infection control, needlestick and sharp injuries, antimicrobial usage density

## Abstract

**Background:**

Healthcare-associated infections (HAIs) are a significant concern in infection prevention. This study analyzes the trend of incidence of HAIs in a tertiary care hospital in China and assesses the effectiveness of cluster based interventions.

**Methods:**

A retrospective analysis was conducted on HAIs reports from 2015 to 2024, focusing on episodes involving the incidence rate of hospital infections, the catheter infection rate related to invasive procedures in the intensive care unit (ICU), healthcare workers’ compliance with hand hygiene, needlestick and sharp injuries (NSIs) among healthcare workers, the prophylactic use rate of antimicrobial agents for Class I surgical incisions, and the antimicrobial usage density (AUD). In 2019, we implemented cluster-based interventions on the incidence of HAIs, strengthening hospital infection control.

**Results:**

The downward trend in HAIs is notable, with infection rates of 9.34 ± 0.25 and 7.29 ± 0.78 per 1,000 patient-days observed during the periods of 2015–2019 and 2020–2024, respectively (*p* < 0.001). The decline in ICU infections linked to invasive ventilators and catheters is evident, with significant reductions in ventilator-associated pneumonia rates per 1,000 ventilator days (6.31 ± 1.50 vs. 2.72 ± 1.01, *p* = 0.002), catheter-associated urinary tract infection rates per 1,000 catheter days (1.66 ± 0.33 vs. 0.99 ± 0.28, *p* = 0.008), and catheter-related bloodstream infection rates per 1,000 catheter days (1.39 ± 0.35 vs. 0.43 ± 0.14, *p* < 0.001) during the periods of 2015–2019 and 2020–2024. A significant enhancement in hand hygiene compliance was observed when comparing the periods from 2015–2019 and 2020–2024, with a statistically significant difference (68.13 ± 3.55 vs. 77.39 ± 3.37, *p* = 0.003). Additionally, a notable decrease in NSIs per 10,000 patient days was observed during the same comparison period, with a statistically significant difference (12.17 ± 1.47 vs. 9.20 ± 1.07, *p* = 0.006).

**Conclusion:**

Cluster-based interventions are effective in reducing healthcare-associated infections in a tertiary care hospital in China.

## Introduction

Healthcare-associated infections (HAIs) represent a significant challenge to patient care and safety. These infections not only elevate the incidence of morbidity and mortality but also incur substantial, potentially preventable healthcare costs and extend the duration of inpatient treatment (1–5). Consequently, HAIs remain a significant public health problem (6). In conjunction with well-established and broadly adopted standard precautions, the majority of HAIs are preventable and can be reduced by up to 70% through the implementation of effective infection prevention and control (IPC) measures (7). However, preventing HAIs cannot be achieved through a single measure, but rather involves multiple aspects of infection control, resulting from cluster-based interventions, which are comprehensive measures. The study indicated that multimodal strategies proved beneficial in enhancing hand hygiene compliance (8, 9). Additionally, several studies have investigated the role and effectiveness of multimodal strategies in reducing catheter-related bloodstream infections (CRBSI) (10). All intervention studies employed a multimodal approach, defining and promoting bundled or comprehensive procedures at multiple levels. Studies focusing on ventilator-associated pneumonia (VAP) demonstrated that multimodal prevention strategies are effective when implemented by a multidisciplinary task force (11). This study retrospectively analyzed the impact of cluster-based interventions, including the establishment and application of a surveillance team of infection control observers, cost reduction for infection control by departments within the hospital, strengthening training, and increasing the number of dedicated infection preventionists (IPs). By considering these cluster-based interventions, we aim to contribute to the existing literature on effective strategies for reducing HAIs and enhancing safety in healthcare environments.

## Materials and methods

The study was conducted over a 10-year period, divided into the phases before intervention (2015–2019) and after intervention (2020–2024).

### Setting

This study was carried out at Guangdong Second Provincial General Hospital, a university-affiliated tertiary hospital located in Guangzhou, Guangdong Province, China, with a bed capacity of approximately 1,730. In 2020, the Infection Prevention and Control team was augmented by two additional full-time staff members, bringing the total number of team members to eight. This staffing level is in accordance with the national standard of one full-time infection preventionist (IP) per 200 beds. The IPC team consists of experts from diverse fields, spanning clinical medicine, nursing, public health, clinical pharmacy, and humanities management. Notably, the team includes four professionals with intermediate titles, one with an associate senior title, and three with full senior titles, reflecting a robust expertise in infection control.

### Infection control observers team

The team of infection control observers was established at the end of 2019, all infection control observers have undergone intensive training. Initially, the primary responsibilities of the observers were to maintain the normal operation of negative pressure isolation wards, supervise the implementation of disinfection, ensure an adequate supply of protective materials, arrange specimen examinations, and alleviate the anxiety of healthcare workers when treating patients, in order to address the airborne transmission in negative pressure isolation wards. This team also plays a significant role in daily operations. In their routine work, they participate in the supervision and management of hospital infection control. They can identify and correct issues in departmental infection control work, as well as potential infection risks in personal protection and operations during medical activities. They also guide the handling of occupational exposure, which can further enhance the quality and safety of medical care provided by healthcare professionals. The IPC team at the hospital regularly conducts relevant knowledge training and assessments for supervisors, and organizes emergency drills on a regular basis to continuously improve the theoretical foundation and capability level of the infection control observers team. The main responsibilities of the infection control observers team differ between emergency periods and non-emergency Period (Supplementary Table S1). To better manage and leverage the infection control observers team, the hospital has established a relevant reward and punishment system in terms of performance. There is a fixed performance-based reward for each person every month. At the end of the year, infection control observers are selected, and corresponding performance rewards are given. If an infection control observers fails to fulfill their duties properly or makes work-related mistakes that lead to hospital-acquired infection incidents or other impacts, the responsibility of the infection control observers will be pursued, and their infection control observers qualification and labor subsidy will be revoked.

### The hospital bears the costs of infection control for clinical departments

Since the end of 2019, the hospital has been responsible for a portion of the infection control costs for clinical departments, including the costs of infection control and prevention supplies, and the costs of vaccinations for occupational exposure to infectious pathogens. The supplies include hand sanitizers, alcohol-free hand disinfectants, and antibacterial hand soaps for healthcare workers’ hand hygiene, disposable disinfectant wipes for surface disinfection, disposable isolation gowns, disposable shoe covers, disposable protective suits, medical protective masks, face shields, and eye shields. We use the average total cost of hand sanitizers, disposable disinfectant wipes, shoe covers, and isolation gowns from the past 3 years in each department as the benchmark value. The hospital’s infection control cost-sharing plan is structured as follows: for costs below the department’s benchmark value, the hospital covers 80% of the expenses, while the department covers 20%. For costs above the benchmark value, both the hospital and the department share the responsibility equally, each bearing 50% of the expenses. Subsequently, every 2 years, the benchmark value of infection control costs will be dynamically adjusted based on the workload of the department.

### Data collection

The data from the Blue Dragon Hospital Infection Real-time Monitoring and Management System (Hunan Blue Dragon Network Technology Co., Ltd., version 6.0) and the Hospital Intelligent Infection Management System (Shanghai Lilian Information Technology Co., Ltd.) are used to collect hospital infection cases. These cases are reviewed and tracked by dedicated infection control specialists in the hospital. For cases of exogenous Healthcare-associated infections, a multidisciplinary discussion and joint ward rounds are conducted, including the diagnosis of hospital infections, the use of antimicrobial drugs, the risk assessment of invasive procedures, the preventive use of antimicrobial drugs, and isolation measures. The final determination of whether a case is a hospital infection is made by two infection control professionals with senior titles. Data on occupational exposure reports from the office automation system of the Affiliated Guangdong Second Provincial General Hospital of Jinan University are obtained. IPs consult and interview the exposed staff to collect demographic information about the affected personnel, which is recorded in the automated office system for easy reference in subsequent risk assessment and measures. Other data are statistically compiled by the information department. Antimicrobial use density (AUD) = antimicrobial dose (g) per period/DDD/number of patients under extended hospitalization during thesame period × 1,000. DDD was defined as the hypothetical mean daily estimated dose for adults for the main indicated illnesses for the medication. Patient-days were defined as the number of nights a hospitalized patient remained in the hospital, calculated based on the number of times their stay extended past midnight. Exclusion criteria for this calculation included individuals discharged without formal admission and newborns in obstetric units who roomed-in with their mothers. The study was approved by the Ethics Committee of The Affiliated Guangdong Second Provincial General Hospital of Jinan University, Guangzhou, China (Approval No. 2025-KY-KZ-102-01).

### Statistical analyses

All statistical analyses were performed using SPSS statistical software, version 29.0. Categorical variables were compared using the chi-square test. The hospital infection rate per 1,000 patient-days was compared between the periods 2015–2019 and 2020–2024 using the chi-square test. Counted data were described by the number of cases (percentage), and the Kolmogorov–Smirnov test was used to verify the normality of the data. For continuous variables that followed a normal distribution, the mean ± standard deviation (SD) was used to describe them, and the differences between the groups were assessed using Student’s *t* test. All statistical analyses were evaluated at the statistical significance level of *p* < 0.05 (two-sided).

## Results

### Trend of healthcare-associated infections

**Figure 1:**
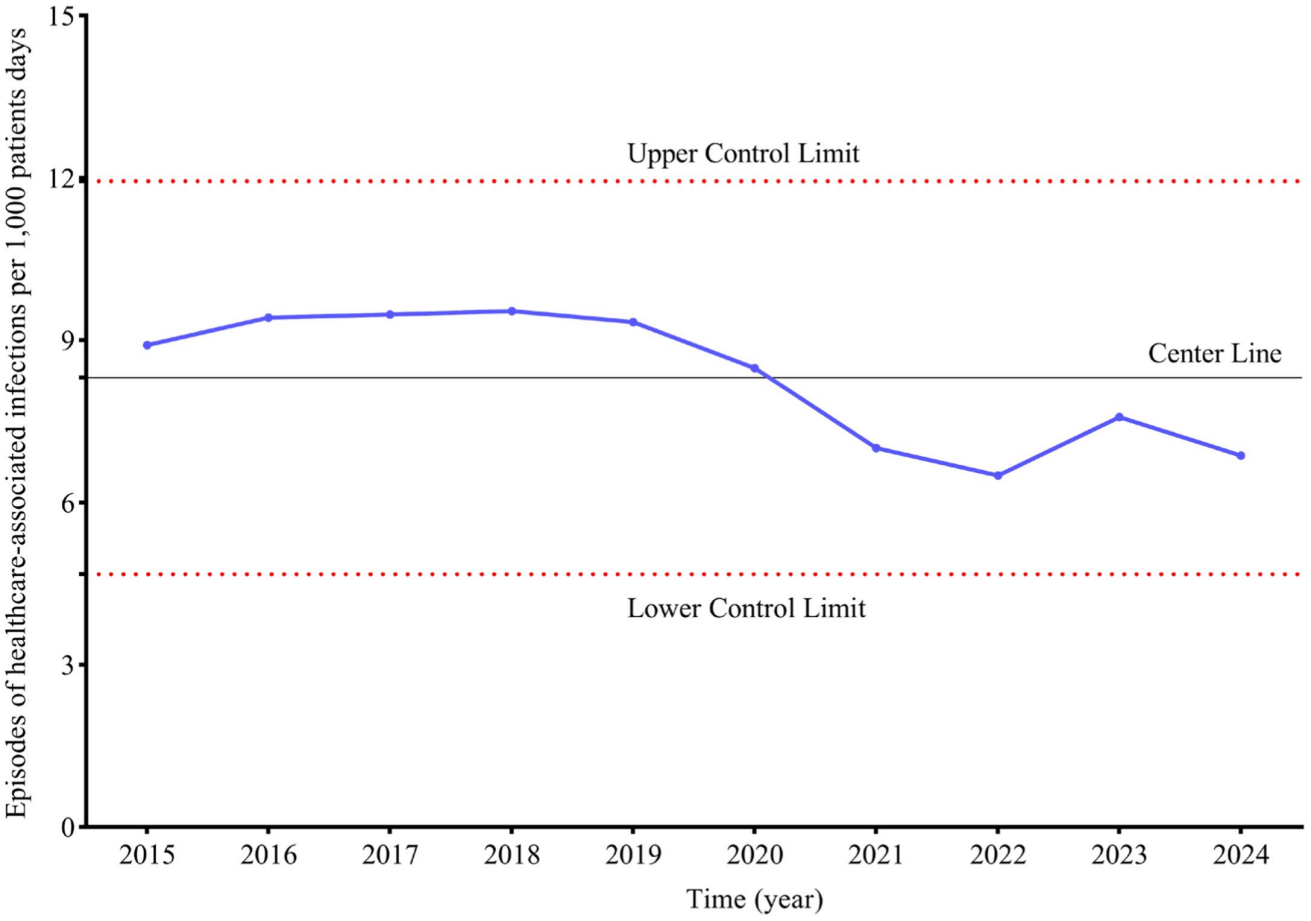
Trend of healthcare-associated infections per 1,000 patients days. Upper control limit is calculated as mean + 3SD. lower control limit is calculated as mean - 3SD.

Between 2015 and 2024, a cumulative total of 5,199 hospitalized patients experienced HAIs. The average number of such infections per year, with a SD, was 520 ± 83. The hospital infection rate, measured per 1,000 patient-days, stood at 8.31 per 1,000 patient-days. When comparing the periods from 2015 to 2019 against 2020 to 2024, the average number of HAIs patients per year, with SD, was 569 ± 36 versus 471 ± 91 (*p* < 0.001). Notably, the hospital infection rate per 1,000 patient-days declined, and this decrease was statistically significant. Specifically, the rates were 9.34 ± 0.25 and 7.29 ± 0.78 per 1,000 patient-days for the periods 2015–2019 and 2020–2024, respectively (*p* < 0.001) (Table 1). The trend in hospital infection rates per 1,000 patient-days from 2015 to 2024 is illustrated in Figure 1 and Supplementary Figure S1.

**Table 1:** Comparing average number of HAIs and hospital infection rate before and after intervention.

Group	Average number of HAIs patients per year	Hospital infection rate per 1,000 patient-days
Before interventions	569 ± 36	9.34 ± 0.25
After interventions	471 ± 91	7.29 ± 0.78
*p*-value	< 0.001	< 0.001

### Trend of intensive care unit infection associated with invasive ventilator and catheters

**Figure 2:**
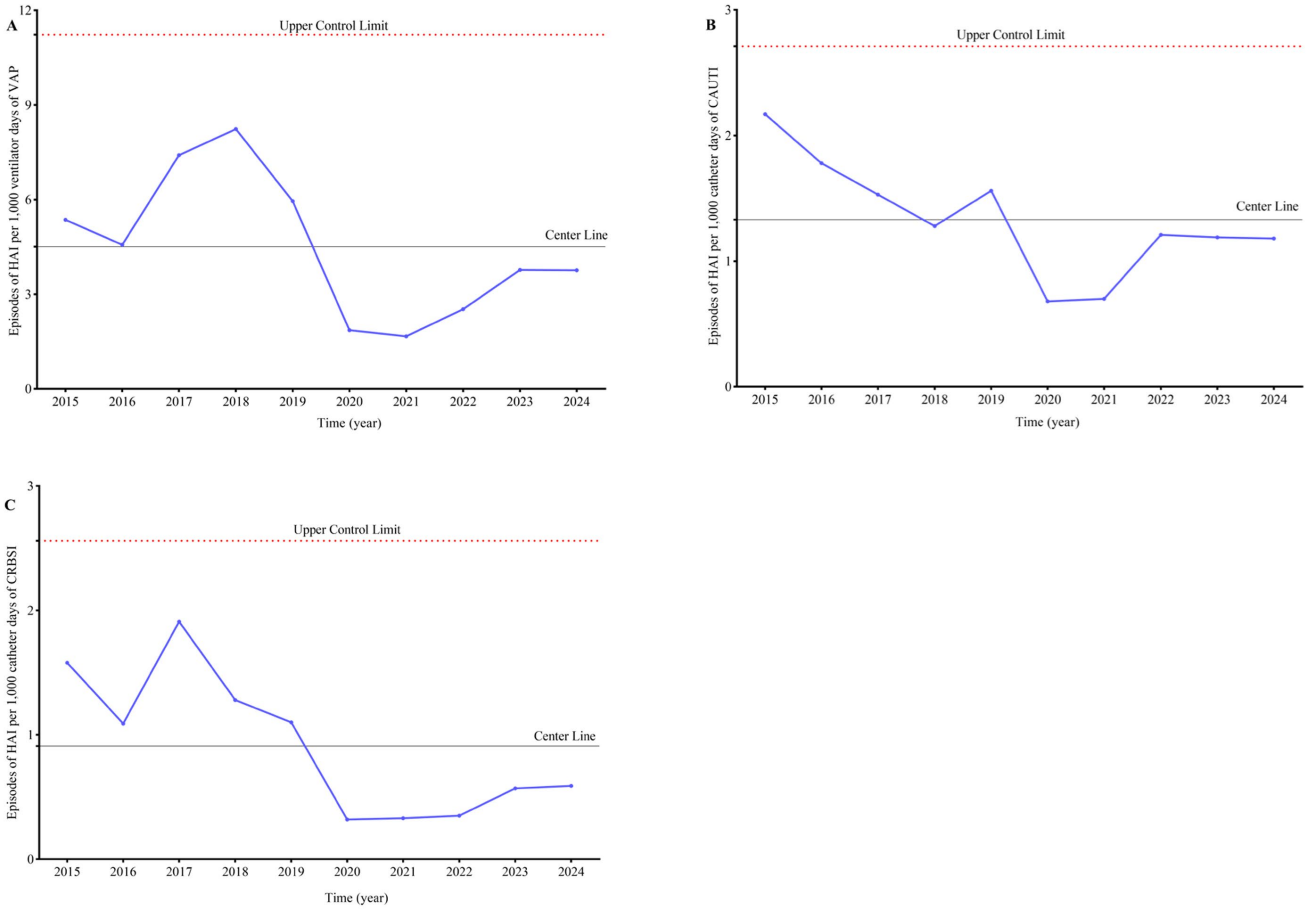
Trend of healthcare-associated infections per 1,000 ventilator/catheter days in ICU. Upper control limit is calculated as mean + 3SD. Lower control limit is set to 0. **(A)** Episodes of HAI per 1,000 ventilator days of VAP. **(B)** Episodes of HAI per 1,000 catheter days of CAUTI. **(C)** Episodes of HAI per 1,000 catheter days of CRBSI.

A comparative analysis of the period from 2015 to 2019 versus 2020 to 2024 revealed a declining trend in the hospital infection rate of ventilator-associated pneumonia (VAP) per 1,000 ventilator days within the Intensive Care Unit (ICU), with a statistically significant difference (6.31 ± 1.50 vs. 2.72 ± 1.01, *p* = 0.002). Similarly, the hospital infection rate of catheter-associated urinary tract infection (CAUTI) per 1,000 catheter days exhibited a downward trend, with a statistically significant difference (1.66 ± 0.33 vs. 0.99 ± 0.28, *p* = 0.008). The hospital infection rate of catheter-related bloodstream infection (CRBSI) per 1,000 catheter days also showed a significant decrease, with a statistically significant difference (1.39 ± 0.35 vs. 0.43 ± 0.14, *p* < 0.001) (Table 2). The trend in hospital infection rates of Episodes per 1,000 ventilator/catheter days in the ICU from 2015 to 2024 is illustrated in Figure 2 and Supplementary Figure S2.

**Table 2:** Comparing hospital infection rate of invasive ventilator and catheters in ICU before and after intervention.

Group	VAP	CAUTI	CRBSI
Before interventions	6.31 ± 1.50	1.66 ± 0.33	1.39 ± 0.35
After interventions	2.72 ± 1.01	0.99 ± 0.28	0.43 ± 0.14
*p*-value	0.002	0.008	< 0.001

### Trends in four hospital infection prevention and control monitoring indicators

**Figure 3:**
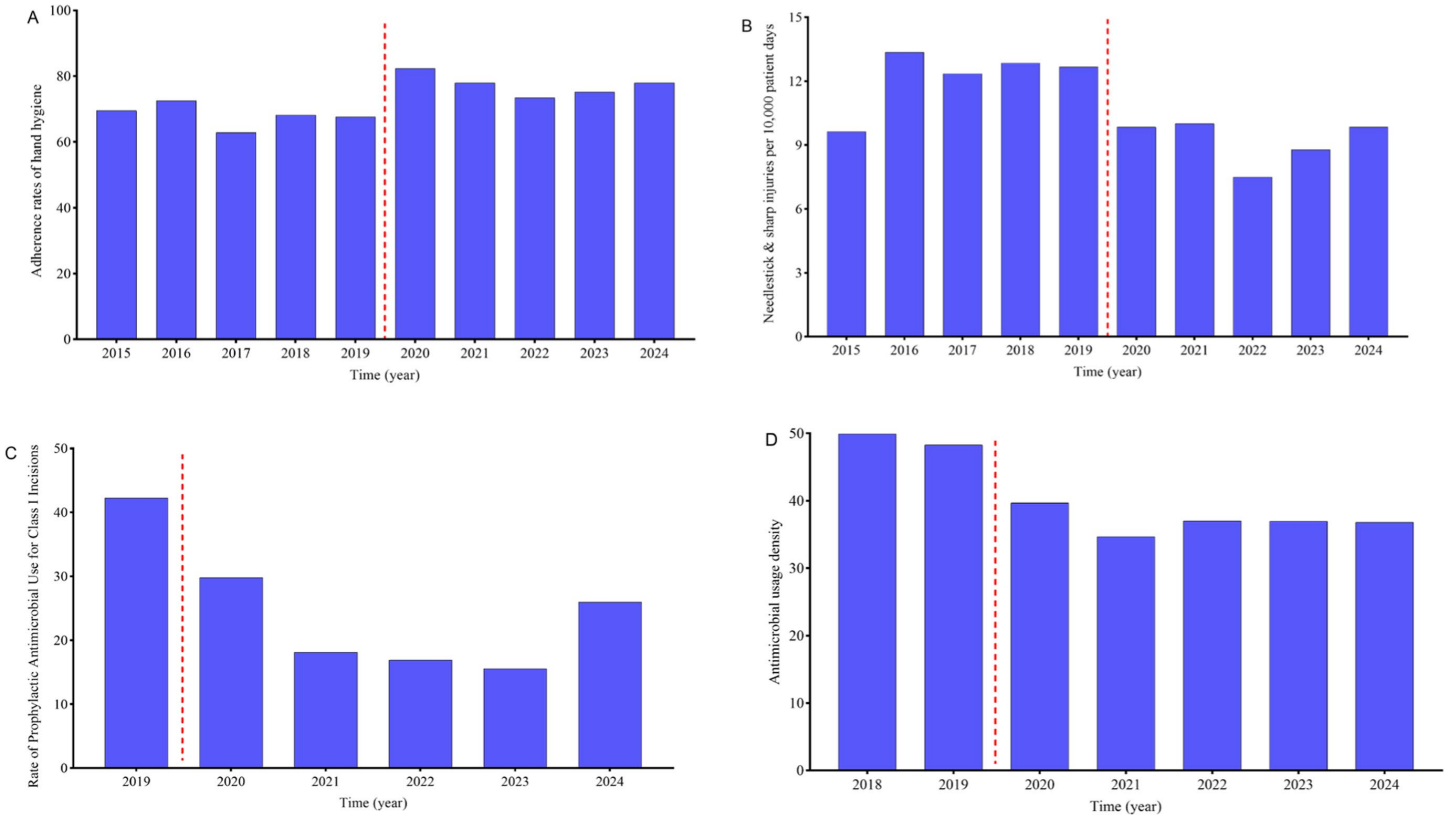
Trends in four hospital infection prevention and control monitoring indicators. **(A)** Compliance with hand hygiene among healthcare workers. **(B)** Needlestick & sharp injuries per 10,000 patient days among healthcare workers. **(C)** Rate of prophylactic antimicrobial use for class I incisions. **(D)** Antimicrobial usage density.

From 2015 to 2024, the mean ± SD hand hygiene compliance rate among healthcare workers was 72.76 ± 5.87. A significant improvement in hand hygiene compliance was observed when comparing the periods from 2015 to 2019 and 2020 to 2024, with a statistically significant difference (68.13 ± 3.55 vs. 77.39 ± 3.37, *p* = 0.003) (Figure 3A). Over the same period, a total of 670 episodes of needlestick & sharp injuries (NSIs) were reported to the infection control team, with an average ± SD of 67 ± 14 episodes per year, equating to an overall mean ± SD of 10.69 ± 1.98 episodes per 10,000 patient days. A notable reduction in NSIs per 10,000 patient days was observed when comparing the periods from 2015 to 2019 and 2020–2024, with a statistically significant difference (12.17 ± 1.47 vs. 9.20 ± 1.07, *p* = 0.006) (Figure 3B). The monitoring of antimicrobial prophylaxis for Class I incisions began in 2019, with a mean ± SD of 24.77 ± 10.23 for the use of antibiotics for prevention of Class I surgical site infection from 2019 to 2024, demonstrating a downward trend over time (Figure 3C). The monitoring of antimicrobial usage density began in 2018, with a mean ± SD of 40.49 ± 6.07 for antimicrobial usage density from 2018 to 2024, showing a downward trend over time (Figure 3D).

### Cluster-based intervention approaches to mitigate infection control risks

**Figure 4:**
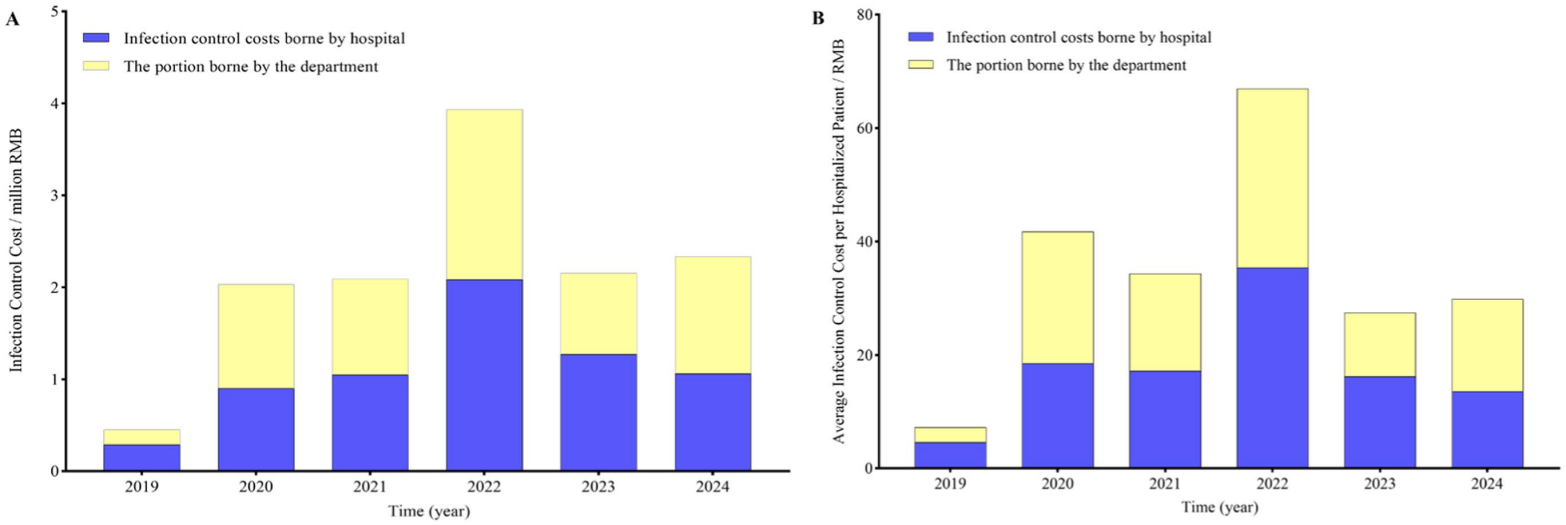
The cost of infection control borne by departments and the hospital. **(A)** The total cost of infection control borne by departments and the hospital. **(B)** The average cost of infection control borne by departments and the hospital per inpatient.

Since the end of 2019, the hospital has initiated a Campaign to Cluster-based intervention approaches aimed at reducing infection control risks, encompassing the establishment of an infection control observation team, the hospital’s partial assumption of infection control costs for clinical departments, an increase in the number of dedicated infection control personnel, and a series of measures including enhanced training and assessment. For the infection control observation team, the average age of junior level is 31.50 ± 3.95 years, whereas that of intermediate level and above is 41.23 ± 6.60 years. In terms of numbers, the majority (*n* = 43, 72.88% of 59) are at the intermediate level and above. Professionally, doctors constitute the majority (79.66%, 47/59). Comparatively, the proportion of surgical departments among junior level and intermediate level and above personnel is 62.50 and 23.26%, respectively, with a statistically significant difference (*p* = 0.013), as shown in Table 3. From 2019 to 2024, the total infection control costs for all departments amounted to 2.17 ± 1.11 million RMB, with departments and the hospital bearing 1.06 ± 0.55 vs. 1.11 ± 0.58 million RMB, respectively (*p* = 0.88). The average infection control cost per inpatient was 34.60 ± 19.59 RMB, with departments and the hospital, respectively, assuming 17.00 ± 9.88 vs. 17.61 ± 10.06 RMB per inpatient (*p* = 0.92), as shown in Figure 4. Furthermore, since 2019, the hospital has intensified training for all levels and types of personnel, increased the frequency of training and assessment, and implemented specialized training programs and content for various levels and types of personnel such as doctors, nurses, and support staff by the infection control team.

**Table 3:** The essential demographic data of the infection control observation team.

Characteristics	Junior level (*n* = 16)	Intermediate level and above (*n* = 43)	*p*-value
Age (mean ± SD)	31.50 ± 3.95	41.23 ± 6.60	< 0.001
Male sex, *n* (%)	7 (43.75)	23 (53.49)	0.51
Occupation, *n* (%)			0.59
Doctor	14 (87.50)	33 (76.74)	
Nurse	1 (6.25)	7 (16.28)	
Other occupations	1 (6.25)	3 (6.98)	
Department, *n* (%)			0.013
Internal Medicine	6 (37.50)	18 (41.86)	
Surgery	10 (62.50)	10 (23.26)	
Other departments	1 (6.25)	15 (34.88)	
Education level, *n* (%)			0.066
Junior or high school and below	2 (12.50)	5 (11.63)	
College degree	3 (18.75)	22 (51.16)	
Postgraduate and above	11 (68.75)	16 (37.21)	

*p*-value derived from chi-square test between the junior level and intermediate level and above group.

## Discussion

The downward trend in HAIs is notable, with infection rates of 9.34 ± 0.25 and 7.29 ± 0.78 per 1,000 patient-days observed during the periods of 2015–2019 and 2020–2024, respectively (*p* < 0.001). Our study summarized the incidence and characteristics of HAIs, infections associated with invasive ventilators and catheters in the ICU, hand hygiene compliance among healthcare workers, NSIs, antimicrobial prophylaxis for Class I incisions, and AUD over a 10 year period. We analyzed the trends for the periods 2015–2019 and 2020–2024, and the comparison between these two timeframes revealed that cluster-based interventions played a significant role in the control of hospital infections. Recent literature on IPs indicates a wide range in staffing ratios, with reported figures varying from as high as one IP per 152 beds (12) to as low as one IP per 69 beds (13). Notably, our hospital achieved a staffing ratio of approximately one IP per 200 beds in 2020, reflecting a commitment to maintaining robust infection control measures despite the challenges associated with resource allocation. Our administrative duties and infection surveillance consistently occupy the top two positions in our workload, accounting for between one-quarter and one-half of our total work hours, in alignment with previous reports (14). Due to the complex interplay of factors involved in infections, establishing a clear cause-and-effect relationship in relation to IP staffing can be challenging. However, the most frequently monitored outcomes in relation to the effectiveness of IP programs include the incidence of CAUTI and CRBSI. Nearly all studies evaluating program efficacy have shown a positive correlation between the utilization of IP professionals and the associated outcome metrics (12, 15–19). Therefore, it is crucial to leverage the expertise of clinical doctors and nurses, and to establish a team of infection control observers. This work underscores the necessity for a novel staffing model that emphasizes a detailed evaluation of each individualized program and care setting. Additionally, it highlights the need for a model that can effectively demonstrate the variability in quantifying the staffing and responsibilities of IP professionals across different settings. Infection control observers have played a crucial role during outbreaks of infectious diseases, such as the COVID-19 pandemic, and continue to be highly effective during non-outbreak periods. They are a practical demonstration of the principle that everyone is an infection control observers (20, 21). For example, hand hygiene is considered the most effective strategy in combating hospital-acquired infections, with studies reporting a significant reduction in infection rates after improving hand hygiene compliance (22). However, currently, it is not very practical for IP personnel to observe hand hygiene compliance among healthcare workers. Instead, having infection control observers from clinical departments to observe and guide hand hygiene compliance can more effectively improve hand hygiene adherence. On the other hand, the investment in infection control costs, particularly the cost of infection control materials such as alcohol-based hand sanitizers, disposable isolation gowns, disposable disinfectant wipes, chlorine-containing disinfectant tablets, and disposable protective suits, if clinical departments bear a significant portion of these costs, it may lead to departments reluctance to obtain and use these materials, resulting in an increased infection rate. Currently, many healthcare institutions are under-resourced, with insufficient reimbursement for infection control measures. This financial constraint can hinder the effective implementation of infection control protocols, as departments may be reluctant to invest in necessary materials such as alcohol-based hand sanitizers, disposable isolation gowns, and chlorine-containing disinfectant tablets. Consequently, this can lead to a higher incidence of infections, as the lack of proper protective equipment and cleaning supplies can compromise the safety and hygiene of patient care environments (23). Ongoing lapses and major errors in infection prevention efforts, including injection safety practices, demonstrate a lack of basic infection prevention practices and processes in many cases. These deficiencies highlight the need for comprehensive training, robust protocols, and continuous monitoring to ensure that healthcare workers are equipped with the knowledge and skills necessary to prevent infections effectively (24). Providing sufficient, easily accessible, and ready-to-use infection control materials such as alcohol-based hand sanitizers, disposable isolation gowns, and chlorine-containing disinfectant tablets is a key element in preventing the transmission of pathogens in healthcare settings. Ensuring that these materials are readily available and properly stocked can significantly enhance the ability of healthcare workers to maintain a high standard of hygiene and safety, thereby reducing the risk of infection for both patients and staff (25). When hospitals assume the infection control costs for clinical departments, healthcare workers are more likely to feel confident in conducting high-risk clinical procedures without undue worry about the availability of infection control supplies. This confidence can mitigate the perceived risk of contamination, enabling medical professionals to concentrate more on patient care and less on the administrative aspects of infection prevention.

This study has several limitations that should be acknowledged. Firstly, it is a single-center study, which may limit the generalizability of the findings to other healthcare settings. Additionally, the retrospective nature of the study involves data collected at different time points, which may be influenced by varying medical practices (e.g., patient severity, bed occupancy rates), policies, and technological advancements at those times, potentially affecting the accuracy of the results. Lastly, the dynamic nature of safety protocols and devices over the study period may impact the consistency of the data, highlighting the need for ongoing research to assess the effectiveness of interventions across diverse healthcare environments.

## Conclusion

Cluster-based interventions have proven effective in reducing HAIs in a tertiary care hospital in China. This underscores the importance of continuous implementation of these strategies to ensure their effectiveness. Ongoing monitoring and assessment of HAIs trends will be crucial for identifying areas that require improvement and for maintaining a safer working environment for all healthcare professionals.

## Data Availability

The raw data supporting the conclusions of this article will be made available by the authors, without undue reservation.
