# Supplementary material for: A comparative analysis of cluster based interventions on healthcare-associated infections in a tertiary care hospital in China
Source: Front Public Health. 2025 Jun 16;13:1599682. doi: 10.3389/fpubh.2025.1599682 (PMC12206888; doi:10.3389/fpubh.2025.1599682)

**Supplementary Material**

Table S1 The duties of an infection control observer

| **Emergency Period** |
| --- |
| 1.Monitor and guide staff in the proper wearing and removal of protective equipment through real-time monitoring systems. Address any issues immediately and make necessary corrections. |
| 2.Instruct medical personnel to adhere to safety protocols and ensure they maintain proper hand hygiene. Supervise these practices to ensure compliance. |
| 3.Observe, supervise, and correct hazardous factors during medical procedures performed by healthcare workers through real-time monitoring systems. If necessary, enter the isolation ward to review the site, streamline processes, and handle emergencies. |
| 4.Monitor the occupational exposure of medical personnel. In the event of an exposure incident, intervene promptly, guide the affected personnel in taking immediate and effective measures, assess the exposure risk, and report it in a timely manner. |
| 5.Maintain constant communication with medical personnel through real-time monitoring systems, paying attention to their behavior and mental well-being. Provide timely support to alleviate their stress and anxiety. |
| 6.Regularly check the negative pressure values in each area of the negative pressure ward, reviewing the airflow diagrams of each negative pressure zone every hour. Report any issues immediately to the engineers. |
| 7.Supervise and ensure the implementation of air, surface, and environmental disinfection, as well as the proper handling of medical waste. |
| 8.During special periods, implement a rotating shift system for infection control observers, with each shift lasting 8 hours and three personnel per day. |
| **Non-Emergency Period** |
| 1.Responsible for supervising and inspecting the protection of medical staff in this department, infection control risk factors, etc. Any issues found should be reported to the department head in a timely manner, along with suggestions for improvement. |
| 2.Responsible for the management and correct use of protective equipment in this department. |
| 3.Supervise medical staff in this department to strictly implement hand hygiene. Represent the management department in participating in hospital-wide covert inspections and cross-checks of hand hygiene, conscientiously fulfilling duties in inspection work, ensuring objectivity and fairness. |
| 4.Supervise the implementation of hospital infection control work in this department, including hospital infections, multidrug-resistant bacterial infections/colonizations, and infectious diseases. |
| 5.Supervise medical staff in this department to strictly enforce aseptic techniques, disinfection procedures, and other rules and regulations. |
| 6.Keep track of hospital infection situations in this department, reminding attending physicians to promptly collect samples for testing before using antibiotics, and to fill out hospital infection report cards within 24 hours. |
| 7.Supervise the implementation of air, surface, and environmental disinfection, as well as medical waste disposal. |
| 8.Assist the Infection Control Department in investigating, identifying infection sources and transmission routes, actively treating patients, and taking effective control measures to prevent hospital infection outbreaks. |
| 9.Ensure the occupational exposure protection of medical staff in this department, assist in filling out the occupational exposure registration form, and follow up on post-exposure management. |
| 10.Assist in the hospital infection management of cleaners, catering staff, caregivers, and visitors. |

Figure S1 Trend of Healthcare-associated infections per 1,000 patients days


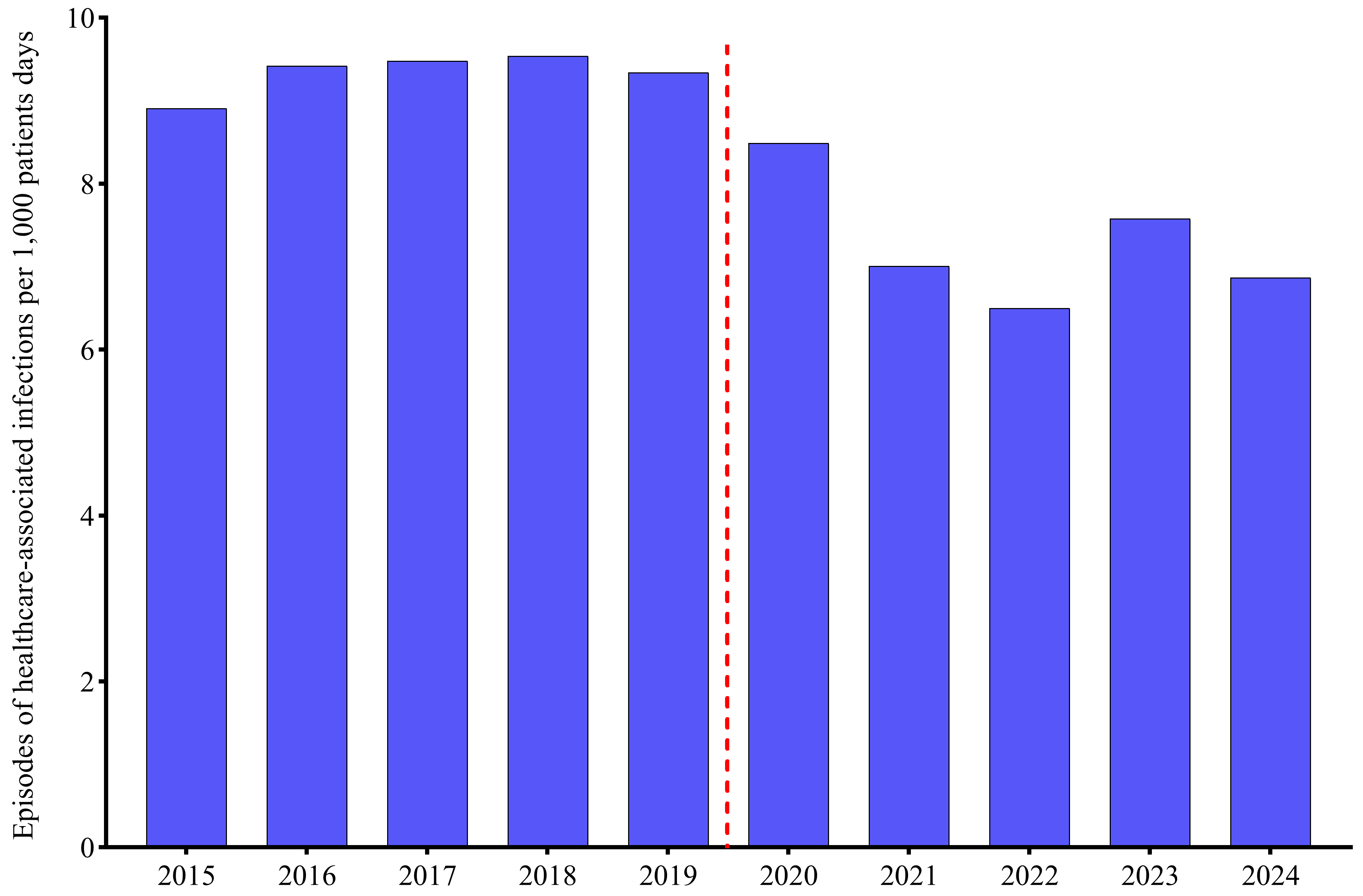


Figure S2 Trend of Healthcare-associated infections per 1,000 ventilator / catheter days in ICU.


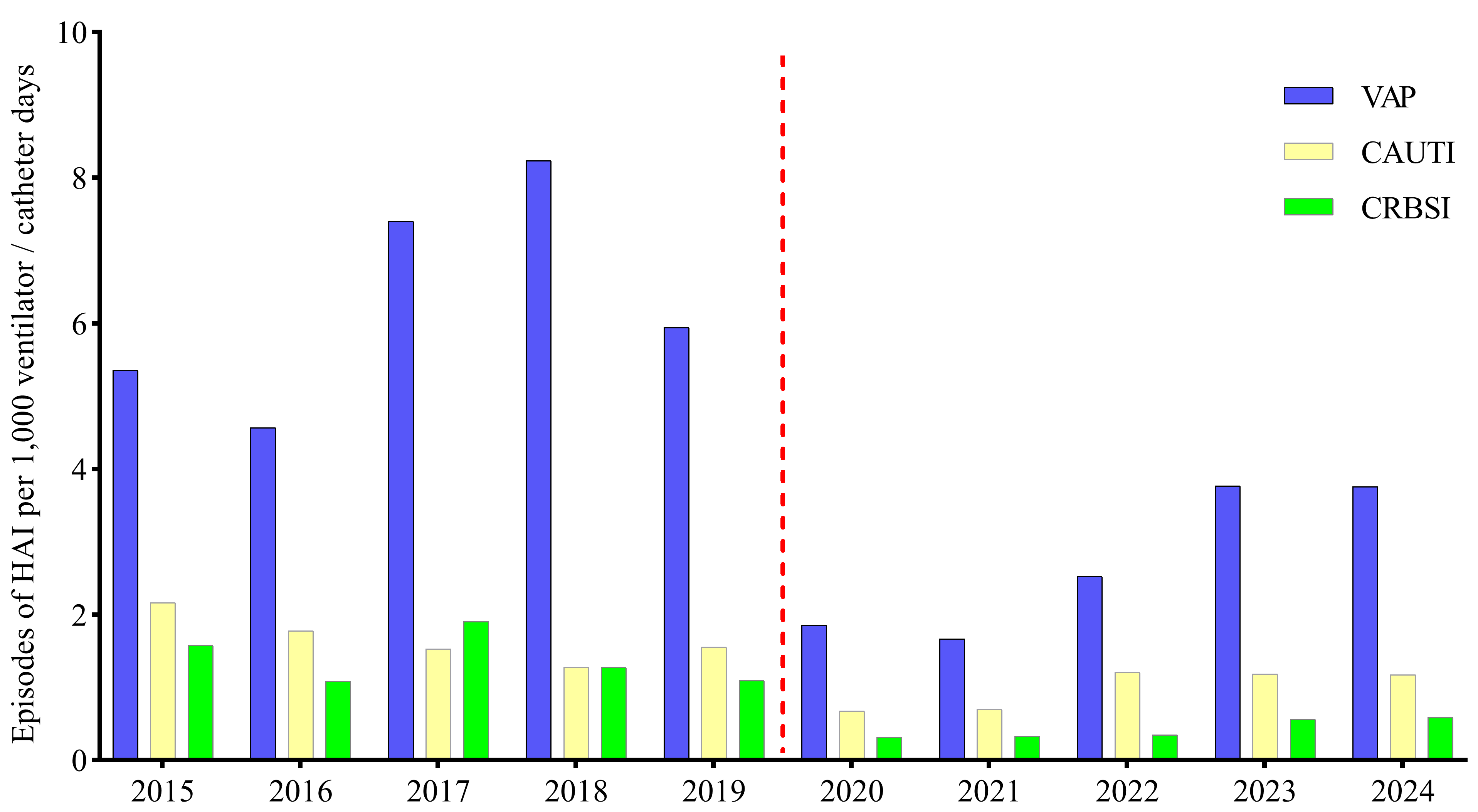

Supplement: Supplementary file 1 [file Data_Sheet_1.doc]
